# Death from Failed Protection? An Evolutionary-Developmental Theory of Sudden Infant Death Syndrome

**DOI:** 10.1007/s12110-024-09474-6

**Published:** 2024-07-29

**Authors:** Herbert Renz-Polster, Peter S. Blair, Helen L. Ball, Oskar G. Jenni, Freia De Bock

**Affiliations:** 1 Division of General Medicine, Center for Preventive Medicine and Digital Health Baden- Württemberg (CPD-BW), University Medicine Mannheim, Heidelberg University, Mannheim, Germany; 2 Department of General Pediatrics, Neonatology and Pediatric Cardiology, University Hospital Düsseldorf, Heinrich-Heine-University, Düsseldorf, Germany; 3 Centre for Academic Child Health, Population Health Sciences, University of Bristol, Bristol, UK; 4 Department of Anthropology, Durham Infancy & Sleep Centre, Durham University, Durham, UK; 5 Child Development Center at the University Children’s Hospital Zurich, University of Zurich, Zurich, Switzerland

**Keywords:** Sudden Infant Death Syndrome, Evolution, Breastfeeding, Evolutionary-developmental theory, Bed-sharing, Prone sleeping

## Abstract

Sudden infant death syndrome (SIDS) has been mainly described from a risk perspective, with a focus on endogenous, exogenous, and temporal risk factors that can interact to facilitate lethal outcomes. Here we discuss the limitations that this risk-based paradigm may have, using two of the major risk factors for SIDS, prone sleep position and bed-sharing, as examples. Based on a multipronged theoretical model encompassing evolutionary theory, developmental biology, and cultural mismatch theory, we conceptualize the vulnerability to SIDS as an imbalance between current physiologic-regulatory demands and current protective abilities on the part of the infant. From this understanding, SIDS appears as a developmental condition in which competencies relevant to self-protection fail to develop appropriately in the future victims. Since all of the protective resources in question are bound to emerge during normal infant development, we contend that SIDS may reflect an evolutionary mismatch situation—a constellation in which certain modern developmental influences may overextend the child’s adaptive (evolutionary) repertoire. We thus argue that SIDS may be better understood if the focus on risk factors is complemented by a deeper appreciation of the protective resources that human infants acquire during their normal development. We extensively analyze this evolutionary-developmental theory against the body of epidemiological and experimental evidence in SIDS research and thereby also address the as-of-yet unresolved question of why breastfeeding may be protective against SIDS.

**Supplementary Information:**

The online version contains supplementary material available at 10.1007/s12110-024-09474-6.

*In memoriam* Remo Largo^†^, teacher, friend, curious companion.

Sudden Infant Death Syndrome (SIDS, or “cot death”) poses the ultimate riddle vexing pediatricians, public health experts, and evolutionary biologists alike: Why should a human infant, after an exquisitely complex intrauterine development, suddenly succumb to death, without any obvious disease to explain the disaster?

With this background it may come as no surprise that both the pathogenesis and the etiology of SIDS are still poorly understood. Environmental (e.g., parental smoking), behavioral (e.g., placing an infant prone for sleep), and social (e.g., social deprivation) risk factors are universally accepted as part of the pathogenetic matrix. The fact that most infants exposed to external risks do *not* suffer adverse outcomes has been explained by assuming additional biological vulnerability on the part of the victims, which may set the stage for deadly exogenous–endogenous interactions under unfavorable temporal conditions (Filiano & Kinney, 1994; Guntheroth & Spiers, 2002). This so-called Triple Risk Hypothesis has been widely accepted as the core theoretical framework of SIDS pathogenesis, although protective influences such as breastfeeding have not been negated (Hauck et al., 2011).

However, there are indications that the prevailing focus on risks may not suffice to explain the pathogenesis of SIDS. A wide range of biological vulnerabilities has been implicated as parts of the pathological matrix of SIDS, yet each of these influences may only be relevant for a subset of cases (Duncan & Byard, 2018). Also, the predictive power of any given risk constellation is dismally poor: among infants with the same risk exposures, only a small minority will succumb to SIDS. Obviously, there is a lot of dark matter in the SIDS universe.

## Unresolved Questions

The dark matter seems to be reflected in the epidemiological record, which presents a wide array of riddles. Risk of SIDS is very low in the first 4 weeks of life and then increases to a five times higher peak between 2 and 4 months of age. As it seems, the younger, more immature infants are relatively protected in a “grace period” of sorts. But why should a 3-month-old baby be more vulnerable to the typical SIDS risk factors than a 3-week-old?

There is more dark matter. Boys are more at risk of SIDS than girls by a margin of 60:40 (Carpenter et al., 2004; Moon et al., 2007). Boys are also more likely to be placed prone by their caregivers (Jonge et al., 1994; Oyen et al., 1997), a position clearly identified as a major risk factor for SIDS. Yet, if one compares the relative risk of SIDS associated with prone sleeping in case-control studies, the individual risk estimate may be up to three times higher for girls than for boys (Oyen et al., 1997). Why should girls be more susceptible to the dangers of the prone position?

To stick with the sex riddle, two thirds of the SIDS victims who die in their own crib are boys (Byard et al., 2012; Fleming et al., 2015). In the bed-sharing situation, however, the gender ratio is equal or even reversed (Byard et al., 2012; Fleming et al., 2015). Could this mean that boys get along better in a shared sleep environment than girls? And if so, how may this be explained?

The shared sleep environment comes with another open question: prone sleeping has been clearly identified as a major risk for SIDS. Yet, prone sleeping may be less predictive of SIDS for routinely bed-sharing infants than for infants sleeping solitarily (Blair et al., 2014; McGarvey et al., 2006).

Conversely, prone sleeping was reported as a strong risk factor for inexperienced prone sleepers—but much less so for routinely prone sleeping infants (Klonoff-Cohen & Edelstein, 1995b; L’Hoir et al., 1998; Li et al., 2003; Mitchell et al., 1999b; Oyen et al., 1997). In an analysis of the Nordic SIDS cohort, 92% of the infants found facing straight down belonged to the group of inexperienced prone sleepers (L’Hoir et al., 1998).

The same experiential background seems to apply to bed-sharing: nonhabitual bed-sharing (“last night only”) was shown to be an important risk factor for SIDS—routine bed-sharing, however, does not seem to be associated with an increased risk of SIDS (Scragg et al., 1993; Vennemann et al., 2012).

## Differential Effects of Risk Factors in Different Infants

Obviously, risk factors alone are not able to explain the epidemiological record of SIDS. Apparently, the “risk currency”—the classic value in SIDS research—has a floating exchange rate. For some infants, a given risk may have a very high value; for others, its worth may be very small—or perhaps even zero.

Here we will argue that this risk moderation may be determined by the *developmental characteristics* of the child. We do so in reference to the behavioral-neurodevelopmental hypothesis formulated by Burns and Lipsitt (1991) and Lipsitt (2003) (partially taken up by Mitchell et al., 1999b, as well as Davies & Gantley, 1994), which we expand and integrate into a wider paradigm which also incorporates evolutionary theory.

This evolutionary-developmental theory, as we call it, conceptualizes SIDS as an outcome at the crossroad between the current environmental challenges that a given infant faces (i.e., his or her risk landscape) and the developmental resources that this infant has accumulated (i.e., his or her protective landscape). We will investigate these protective variables in detail, describe their evolutionary origin, and will argue with reference to the existing literature how this evolutionary-developmental theory may provide an understanding of SIDS beyond the predominantly risk-based hypotheses.

As an example, we will examine the arguably most significant risk factor for SIDS and accidental suffocation, prone sleeping, which in many countries has been attributed to half or more cases (Mitchell & Thompson, 2001; Oyen et al., 1997; Priyadarshi et al., 2022; Trachtenberg et al., 2012). We will present evidence that the risk associated with prone sleeping fluctuates with the presence of modifying influences and may in fact be yet another exemplar of the floating exchange rate suggested above: instead of exerting a “fixed” risk effect, prone sleeping may constitute a facilitating risk influence under specific conditions.

We will then expand on other risk determinants, including exposure to tobacco smoke and unsafe sleep environments. Finally, we will discuss how the evolutionary-developmental theory could help clarify the—as of yet unexplained—protective effects of breastfeeding and how it may fit with recent suggestions that bed-sharing under safe conditions may be protective against SIDS (Bartick et al., 2022; Blair et al., 2014).

## The Developmental Perspective

### The Riddle of the “Grace Period”

… even the smallest and weakest of babies have no trouble at all in squirming out from under unwanted coverings. All parents know that well (Raring, 1975:63)^1^

As noted above, the epidemiologic profile of SIDS follows a peculiar temporal pattern, with an apparent “grace period” of a comparatively low incidence covering the first developmental stage, the newborn period—a pattern that is also observed for accidental suffocation (Erck Lambert et al., 2019; Shapiro-Mendoza et al., 2006). This pattern is very different from the mortality distribution in infant deaths in general, which goes along with postnatal maturity: the more immature the child, the higher his or her risk of death. Indeed, SIDS and accidental suffocation are the only infant “disorders” that to a certain extent spare the neonate (Guntheroth & Spiers, 2002). This postnatal relative benignity is followed by a brisk increase in risk for SIDS in the second month, reaching a peak incidence between 10 and 24 weeks of age, when 85% of SIDS deaths occur.

The question why there may be an increased vulnerability in the 2- to 5-month bracket has inspired several hypotheses (Blackwell et al., 2015; Fleming et al., 1994; Guntheroth & Kawabori, 1975; Spiers, 2000). None of them has been more thoroughly drawn up and bolstered by experimental studies than the behavioral-developmental theory of Lewis Lipsitt (in part worked out together with Barbara Burns; Burns & Lipsitt, 1991; Lipsitt, 1976, 2003). As it happened, their focus (now more than 50 years ago) was prone sleeping and the very question: what processes may make prone sleeping a risk that appears to surface much more strongly *after* the neonatal period? Burns and Lipsitt used this question to develop a distinct “behavioral” hypothesis of SIDS which claims that infants may depend on some sort of *learned protective behavior* to stay safe once they exit their first developmental period, during which they had been protected by innate reflexes.

Their idea started from Myrtle McGraw’s description of a biphasic infant development, which she exemplified with studies of the grasping, stepping, and diving reflex (McGraw, 1963). Initially infants rely on their inborn subcortical protective behaviors to protect themselves against environmental challenges, such as immersion in water or occlusion of the airways (type A behavior). After the neonatal period this reflectory program gradually subsides to allow for the flexible development of volitional regulatory and motor competences which finally supplant the initial reflectory program, with volitional regulation fully established by about 5 months of life (type C behavior). In between, infants go through a critical or “disorganized” transition period in which their innate protective repertoire is gradually supplemented by the emerging volitional responses (type B behavior). How closely this behavioral sequence mirrors neurological maturation and how much it may relate to the infant’s capacity for learning of protective behaviors can be gleaned from observations in premature infants, where disturbed type A and type B patterns can indicate a risk of cerebral palsy. In fact, reduced general movement variability (manifested in abnormal type A and B behaviors) can be used as a clinical indicator of disturbed learning capacities in premature infants (De Bock et al., 2017; Groen et al., 2005).

Importantly, and central to the “behavioral” theory of SIDS by Burns and Lipsitt, these volitional, “cortical” responses do not appear automatically but rely on learning, through which the infants gradually augment, upgrade, and, finally, replace their inborn capacities (Burns & Lipsitt, 1991; McGraw, 1963). More specifically, this learning relies on two components: biological readiness and environmental support. This means that, for the development of an adequate protective volitional response to occur, infants must have sufficient biological resources but also need to be provided with a social and physical environment conducive to *learning through experience.*

## Transitional Challenges—and Their Solution

Burns and Lipsitt (1991) have identified this transitional learning period as the ultimate ground zero for SIDS. Referring to the complex challenges of breathing and airway control, they argue that infants become vulnerable to SIDS if they cannot muster the transition from subcortical reflexive behaviors to learned cortical behaviors in an adequate and timely manner—whether for lack of learning opportunities or because of preexisting neurodevelopmental limitations.

The most pertinent example may be the infant’s response to airway compromise (for a review, see Thach, 2018). Any covering of the neonate’s nostrils or mouth elicits a fixed order of reactions: First, the baby shakes its head from side to side, then pulls its head back (head lift), moves its hands to the face, and then, if its airways are still compromised, starts to cry and thrusts the covering object away from its face, optionally followed by a sigh of relief (Swift & Emery, 1973). However, as Swift and Emery showed in their experiments with nasal occlusion, this response differs in intensity and effectiveness between infants. Interestingly, airway protection was more effective in newborns than in older infants, suggesting waning reflectory abilities and supporting McGraw’s assumptions of a biphasic developmental model (Lipsitt, 2003; Swift & Emery, 1973). As Burns and Lipsitt spell out in detail, this acquisition of novel, volitional protective abilities relies on two key ingredients: biological readiness and environmental opportunities for learning (Burns & Lipsitt, 1991; Lipsitt, 2003).

The importance of biological readiness has been underscored by observations by Anderson and Rosenblith, who, in the 1960s, examined the respiratory occlusion reflex of newborns and found that infants with a weaker reflectory response were more likely to subsequently succumb to SIDS (Anderson & Rosenblith, 1971). Possibly, these infants enter the above-mentioned transition period at a lower learning level or with distinct impediments relevant to learning, and may thus remain more vulnerable.

Studies of neonatal learning undertaken as early as the 1920s have elucidated how infants expand their inborn reflex repertoire and how heavily this relies on associative learning (operant conditioning; for a review, see Tarullo et al., 2011, and Lipsitt, 1998). According to this body of experimental work, both approach behaviors (such as rooting or sucking) and escape behaviors (such as head turning) are readily reinforced through sensory and social cues from the baby’s normal environment. For example, human infants rapidly learn to alter their sucking behavior in response to the sound of their mother’s voice (DeCasper & Fifer, 1980). They also rapidly learn to associate the exposure to citrus odor with head turning while being gently stroked—a learned response that they remember for at least 24 h and which can then be triggered by the odor alone, even during sleep (Sullivan et al., 1991). Other experiments using air puffs or other stimuli have shown how easily neonates can learn to change their behavior in response to environmental cues, whether awake or asleep (Fifer et al., 2010; Siqueland & Lipsitt, 1966).

Paluszynska et al. (2004) as well as Lijowska et al. (1997) have shown in experiments how relevant such learning may be for infants to manage the challenges of the prone position. As they show, infants placed prone easily encounter situations of airflow restrictions and must then escape suffocation by complex coping behaviors, including both motor and regulatory competences related to arousal management and adequate signaling. When experimentally subjected to respiratory compromise (either by covering their faces or by exposing them to higher carbon dioxide levels), the prone infants react with motor strategies such as “nuzzling,” lifting their heads, and/or turning their heads to one side or the other, accompanied or followed by sighs, startles, limb thrashing, and finally full arousal and signaling (crying). As both Paluszynska et al. and Lijowska et al. show, these strategies are very differently developed and combined and thus differently effective between infants. Turning the head to *both* sides, for example, was typical for experienced prone sleepers only (who, in Paluszynska’s study, also had advanced gross motor development compared with inexperienced infants). Interestingly, in both studies, neither the conceptional age nor the actual age of the infants correlated with the efficacy of the protective behaviors observed, again suggesting experience as the modulating influence (Paluszynska et al., 2004).

This adds to other evidence for a role of learned behaviors during the prone sleeping experience. The prone sleeping infant can frequently be observed in a face-straight-down position—indeed, this seems to be a common occurrence in normal infants 10 to 22 weeks of age studied sleeping prone (Waters et al., 1996). These episodes—which apparently trigger brief episodes of asphyxia due to rebreathing expired air—were shown to have a median duration of more than 3 min but are invariably terminated by brief arousals followed by the infants’ active movements, which include turning the head from one side to the other (Chiodini & Thach, 1993; Waters et al., 1996).

## The Lesson of Breastfeeding

Early physiologists have described similar “learning by doing” in their observation of infant behavior during breastfeeding (Gunther, 1961; Lipsitt, 1998). Not surprisingly, babies frequently experience restricted breathing while at the breast, to which they react with protective maneuvers such as turning their heads. As it seems, these maneuvers—being heavily reinforced through the resulting increase in airflow—get more effective through exposure. Indeed, babies apparently show signs of improved reactions after just one or two experiences of nasal occlusion during breastfeeding (Gunther, 1961). Here again, learning from experience within a “normal,” expectable care environment may play a central role—and the complete lack of such “learning opportunities” during bottle-feeding may come to mind. Experiential learning of protective behavior at the breast may indeed be a promising explanation for the protective effects of breastfeeding against SIDS (to be discussed in the section on “The Breastfeeding Riddle,” below).

Interestingly, animal experiments of exposure to hypoxia during quiet sleep show what may happen if such associative learning is not allowed for. Instead of learning how to protect themselves, rat pups show habituation (decreased responsiveness)—in other words, their hypoxia-induced arousal response gets weaker (Darnall et al., 2010). This may be highly relevant to the human infant, too. Infants with developmental risks may be less able to mount an effective escape response in situations of airflow compromise and be less likely to experience “reward” from increased airflow. This may induce a vicious cycle: instead of learning to escape, the infant may habituate—and become more vulnerable once faced with severe or long-lasting events of airway obstruction (Tarullo et al., 2011).

## Epidemiological Support for the Developmental Perspective

The epidemiological record exemplifies the heavy weight that developmental readiness may carry in relation to the current environmental risks an infant is exposed to. As early as 1979, Lipsitt, Sturner and Burke published a case-control study of SIDS victims within their cohort of 4000 infants they followed longitudinally. They found that the variables that distinguished SIDS victims and survivors were all indicators of what they termed “early-onset developmental impairment” (Lipsitt et al., 1979). These neurodevelopmental risks included low APGAR scores, low birth weight, postnatal respiratory distress, and prolonged hospital stays—a collection which made the authors state: “Perinatal distress parameters, combined with sensorimotor and learning indices in the newborn, may well provide the sharpest definition of those infants most vulnerable to SIDS” (Lipsitt et al., 1979). Incidentally, the same perinatal parameters are shown to go along with general movement restrictions—abnormal type A and B behaviors as described above (Mallmann et al., 2023).

Since then it has become apparent that the vast majority of SIDS victims have experienced suboptimal intrauterine conditions (Blair et al., 2006b; Oyen et al., 1995), and these seem to multiply the effects of the environmental risks encountered (Oyen et al., 1997). In the Nordic Epidemiological SIDS Study, for example, the odds ratio for SIDS for prone-sleeping infants with a birth weight of > 2500 g was 11.6 [95% CI: 6.8–20]; for infants < 2500 g, it was 83.2 [95% CI: 25–276] (Oyen et al., 1997). This fits with observations that infants with low body weight from any cause have a maturational delay in their development of protective head turning (Ratliff-Schaub et al., 2001; Waters et al., 1996).

Plausibly, the developmental disadvantage may also be reflected in the position of SIDS victims at the time of death. In the New Zealand Cot Death Study, in the group of SIDS victims who died in the face-down position, the proportion of infants with a birth weight under 2500 g was 3.5 times higher than in the group of SIDS victims not face down at the time of death (Thompson et al., 2006).

Although this shows that biological factors like prematurity, low birth weight, or exposure to tobacco toxins during pregnancy may set up a “developmental bottleneck” in SIDS pathogenesis, there is evidence that postnatally acquired developmental risks may also contribute—and may become more influential under unfavorable biological preconditions. In the Nordic Epidemiological SIDS Study, 92% of the SIDS victims found facing straight down belonged to the group of inexperienced prone sleepers (L’Hoir et al., 1998). Similarly, Mitchell, in his analysis of the New Zealand cot death cohort, found only 10% experienced prone sleepers among those SIDS victims who turned to the prone position during their last sleep (Mitchell et al., 1999b).

How closely SIDS risk from prone sleeping may be tied to experience-based competencies acquired during development has also been noted in other observations. In the New Zealand cot death study, infants who have established a pattern of spontaneously changing sleep position (either turning from or to prone) were at much lower risk of SIDS than infants without these abilities (Mitchell et al., 1999b). The same was observed in studies from Scotland and from the Netherlands (Brooke et al., 1997; L’Hoir et al., 1998).

Why the risk of succumbing to SIDS in the prone position is so closely tied to developmental readiness may find an explanation in the higher physiologic challenges of the prone position. Several regulatory systems are under higher strain in the prone position, including blood pressure regulation, temperature regulation, brain perfusion, and cerebral oxygenation as well as autonomic control of arousal and cardio-respiratory responses (Chiodini & Thach, 1993; Galland et al., 2002; Yiallourou et al., 2008; for a review, see Horne, 2018). It is therefore plausible that especially the infants with developmental impairments and thus inadequate protective skills will benefit most from being placed supine—in a position in which the airways are more reliably protected from blockage (Lipsitt, 1976; Swift & Emery, 1973).

Finally, the evolutionary-developmental theory may be able to explain the somewhat counterintuitive finding that premature infants tend to die of SIDS at a later (uncorrected) postnatal age than term infants, with the most premature infants showing the latest SIDS mortality peak (Habich et al., 2024). This extension of the neonatal “grace period” in the more immature infants could relate to the fact that primitive reflexes seem to disappear later in premature infants (Olhweiler et al., 2005), possibly indicating a later onset of the “disorganized” transition period described by McGraw.

## Which Abilities Exactly?

The assumption of the prone position as the physiologically more challenging sleep position can explain why the risk of SIDS is so intricately tied to the sleep position. Evidently, infants placed primarily supine cannot turn to prone on their own in the first few months of life and are thus protected from any dangers that may be associated with the prone position (the timing of being able to roll over varies widely between individual infants, ethnicities, sex, and routine sleep position; for a review, see Nelson et al., 2004). It is therefore plausible that the acquired skill of rolling over may contribute to the peak incidence of SIDS in the 2- to 5-month bracket. Yet, protective abilities independent of the ability to actively change body position completely must also play into the positional risk. For one, only a tiny proportion of infants suffers lethal consequences from rolling over—most infants have enough protective abilities to be resilient to the prone position. Also, when infants are being put to sleep in the prone position in the first place, their risk of SIDS varies greatly by age, with infants 1 to 12 weeks of age about four times less likely to experience SIDS than the 13- to 24-week-old infants (Oyen et al., 1997). While this could reflect the changing prevalence of exposure at different ages as well as interaction with other age-related factors, it may also indicate greater positional resilience in the younger infants.

Identifying exactly which abilities may help prone-sleeping infants to protect themselves adequately remains speculative. Yet, the search for an answer may benefit from a closer look at the cascade preceding SIDS. A significant portion of SIDS victims apparently move within their sleep spaces “in such a manner as to allow their faces and/or heads to become covered by bed clothing” (Thach, 2001). Another substantial number rolled into a secondary prone position without entrapment or covering and then died. Apparently, infants either fall or are pushed into the prone position passively (e.g., from the inherently unstable side position), or they may use their motor abilities as a first step to maneuver themselves into potentially dangerous zones—but then some of them may lack the motor or regulatory abilities to cope with the situation (e.g., protect their airway adequately) or to steer clear of the danger zone. The first step may be easier than the second one or may not need much learning. The second step(s), however, may require competencies reinforced through practice. As Paluszynska et al. (2004) show, turning out of the face-down position is an actively learned skill, and it is only perfected after the infant has acquired the ability to turn its face down. As also described by this team, prone-sleeping infants of any age frequently turn their heads into the face-down position (facing the sleep surface directly). How well they then can react in case of compromised breathing seems to depend on experience: the inexperienced infants will only nuzzle and lift their heads. However, truly effective airway management relies on more complex behaviors such as turning the head, ideally to both sides—an ability only found in the experienced prone sleepers in their study. This, again, may be a reminder that infants learn through associative learning how to “expand” their reflective head lift into more complex abilities.

There has been a lot of debate about the role of inadequate arousals in explaining the increased risk of SIDS in prone-sleeping infants. As of yet, there is no clear pathophysiological mechanism to explain the association (Fleming et al., 2017). Although prone sleeping may increase the arousal threshold relative to supine sleeping (Hauck & Hunt, 2000; Horne et al., 2001), there is no indication that an “arousal deficit” alone—independent of other developmental or regulatory deficits—can explain the increased risk of prone sleeping (Thach, 2001). Physiologically, the development of autonomous regulation (including cardiorespiratory control and arousability) and general cortical status are closely interlinked (Kahn et al., 2003). In the same vein, neuropathological studies of SIDS victims have shown pervasive deficits in multiple regulatory brain regions in the brainstem and cerebellum, all involved in respiratory regulation, cardiovascular control, as well as arousal (Horne, 2018). Likewise, SIDS victims were found to experience reduced or incomplete arousals (Kato et al., 2003) but also to move less during sleep, (Kahn et al., 1992; Schechtman et al., 1992), have lower stamina and activity levels (Burns & Lipsitt, 1991; Kaada, 1994), as well as live in an environment with lower estimates of developmental stimulation (Kelmanson, 1993). So, plausibly, the increased risk that the prone position obviously confers to some infants may be related not just to inadequate arousal once compromised but rather to neurodevelopmental risks pertaining to the whole chain of events in the respiratory defense response, which includes both regulatory and motor abilities.

## Toward a Common Denominator: How to Explain Variations on the Risk of Prone Sleeping

The above observations describing the importance of developmental factors for safe sleep in the prone position should, of course, be reflected in the epidemiological record. Indeed, the association between SIDS and prone sleeping varies with the presence of a range of influences, which mirror (a) increased environmental challenges and (b) decreased developmental readiness.

The risk of SIDS associated with the prone position was shown to be higher in winter, in colder latitudes, or if the infant is overheated or ill (Mitchell et al., 1999a; Ponsonby et al., 1993). Increased risk is also associated with a vast array of physical properties of the infant’s sleep space, such as heavy bedding or a soft or uneven sleeping surface (Kemp et al., 1994; Ponsonby et al., 1993). These influences may all be summarized *as increased current adaptive challenges* since they pose increased regulatory demand on the protective abilities of the infant.

Other risk associations of prone sleeping appear to be developmental in nature and seem to reflect *maturational readiness*, as evidenced by.

- *the age factor*: prone sleeping is evidently more dangerous between 2 and 5 months of age (Oyen et al., 1997). As suggested above, this may in part be explained by inadequately developed volitional protective strategies as infants lose their inborn (fully reflective) protective repertoire which had provided them with some protection during the neonatal phase.
- *The influence of intrauterine neurodevelopmental risks*: prone sleeping was shown to be more dangerous for infants exposed to tobacco toxins in utero, born prematurely, or small for gestational age (Oyen et al., 1997). These influences again may be markers for inadequate development of protective abilities due to neurodevelopmental risks. Interestingly, prematurity and low birth weight alone may be less influential on the risk of SIDS than exposure to intrauterine tobacco toxins, which were shown to have pervasive effects on brainstem function (Schellscheidt et al., 1997).
- *The experience factor*: prone sleeping was shown to be more dangerous for infants inexperienced in prone sleeping (Klonoff-Cohen & Edelstein, 1995b; L’Hoir et al., 1998; Mitchell et al., 1999b). This also may be best explained by truncated developmental experiences: a lack of learning opportunities may not allow for full protective capacities to develop.
- *The influence of sex*: on the individual level, prone sleeping is reported to be distinctly more dangerous for girls than for boys (Oyen et al., 1997). This, too, could be explained by the experiential factor. In everyday life, male infants seem to be routinely placed in the prone position for sleep distinctly more often than girls (Blair & Ball, 2004; Jonge et al., 1994; Ottolini et al., 1999; Oyen et al., 1997). Given that the parents’ motivation to place an infant prone usually revolves around soothing (Brenner, 1998; Colson et al., 2017; Moon & Omron, 2002; Ottolini et al., 1999), it may be assumed that prone placement in many instances already begins in the neonatal period. The more intense—and possibly early—experience with prone sleeping may provide the male infant with an experiential head start relative to girls and may thus reduce the individual risk of prone sleeping for boys. Indeed, boys were shown to turn to prone from another sleeping position earlier and about three times more often than girls, indicating a higher level of motor competence, which may be genetic/biological in nature or acquired through experience (Capute et al., 1985; L’Hoir et al., 1998).
- *The influence of the sleep environment*: prone sleeping may be associated with a higher risk of SIDS for solitary sleeping infants than for bed-sharing infants (Blair et al., 2014; McGarvey et al., 2006). This difference in relative risk of being placed prone may be explained in three ways: (i) the bed-sharing infants as a group may be able to rely on a comparatively better developmental readiness since they are frequently—and right from the beginning of life—raised in proximal, “interactional,” and thus developmentally fostering care environments (to be expanded on later); (ii) the intense co-regulatory interaction while bed-sharing may provide the infant with external regulatory support from the mother to better master any challenges which may pose increased regulatory demands, such as prone sleeping; and (iii) the bed-sharing environment engages different infant care practices than the solitary environment, most notably the readiness to breastfeed where the infant is more likely to be placed supine or in a stable side position rather than prone.
- *The influence of day versus nighttime*: prone sleeping seems to be more dangerous during daytime (Blair et al., 2006a, b; Williams et al., 2002). The same has been observed for side sleeping, which may be a nonsignificant influence during night sleep, yet strongly associated with risk during daytime sleep (Blair et al., 2006a, b). It can be speculated that infants are more likely to encounter adverse external circumstances during daytime naps—such as lacking supervision or being placed on a sofa or being placed prone without prior experience by a non-caregiver. Alternatively, they may be more likely to lack the intuitive co-regulatory support of their (bed-sharing) caregiver during naps that they may receive during night sleep (a similar loss of co-regulatory support may happen if infants are placed in a separate room for naps but accustomed to sleeping in the parents’ room for night sleep).

To summarize, the risk of prone sleeping seems to relate to two separate influences: developmental readiness on the one hand and current environmental challenges on the other hand. These influences interact intricately, and this interaction resonates in the outcome: the more unfavorable the balance between the two, the higher the risk of SIDS seems to be. Thus, the common denominator of all the risk associations around prone sleeping may be best described as a *mismatch between the current external challenges that a baby encounters and the current state of its developmental protective abilities.*

How heavily the developmental factor contributes to this equation can be gleaned from the fact that SIDS victims, in their vast majority, are among the most developmentally disadvantaged infants. They are either subjected to biological neurodevelopmental risks (such as intrauterine growth restriction, preterm birth, or intrauterine or extrauterine exposure to drugs or tobacco toxins) or they are subjected to social handicaps—for example, a care environment that may not allow for sufficient learning to happen (both influences may explain the much higher prevalence of SIDS in families with low socioeconomic status, where biological risks and low levels of developmental stimulation often meet) (Burns & Lipsitt, 1991; Kelmanson, 1996a, b). The developmental risks described here may go unnoticed for most babies but may become consequential once a baby is faced with increased adaptive challenges such as heavy or loose bedding, being placed in an unaccustomed sleep position, being exposed to bed-sharing without prior experience, being exposed to a soft sleeping surface, elevated ambient temperature, environmental tobacco smoke, lack of co-regulatory input (e.g., while sleeping in a separate room), low-grade infections, or other biological vulnerabilities.

## The Evolutionary Perspective

As we have seen, the development of protective abilities—central to the avoidance of SIDS—may be truncated by biological constraints or by a care environment not conducive to developmental learning. Here we want to focus on the latter and ask a pivotal question: *Through which experiences* do human infants develop their protective abilities? In other words: *Which care environment* may be appropriate for sufficient protective experience to be accumulated? Obviously, this question is paramount for the design of preventive measures. Incidentally, the question has been explored in SIDS research in the debate about the safety of bed-sharing (McKenna, 1990a, 1990b; McKenna & Mosko, 1990). Yet, a discussion of a possibly “protective” infant care environment in general in regards to SIDS is still missing.

Clearly, this discussion leads away from cultural norms and prescriptions and instead focuses on the deeper underpinnings of human development—its evolutionary biological trajectories. After all, the human infant’s developmental strategies have been calibrated in the environments encountered across our species’ history, the so-called environment of evolutionary adaptedness (Bennett, 2018). To this day, children acquire their protective potential by maturing along the developmental trajectories established in the evolutionary process.

## The “Evolutionary Care Package,” Its Maturational Implications, and Its Ramifications for Autonomous Control

Human infants, being the most developmentally immature of all precocial mammals, develop within a close co-regulatory context with their caregivers. The care received can be viewed as a “package” of four ingredients (described in Konner, 2010): breastfeeding, bed-sharing, extensive parent-baby contact, and baby transport on or at the caregiver’s body.

All of these evolutionarily expected exposures (which have also been summarized as “proximal” infant care) (St James-Roberts et al., 2006) have clear effects on infant development in general and the acquisition of protective developmental skills in particular. Most interestingly, there appears to be a close link from this evolved experiential “package” to the possibly most central hub of SIDS pathogenesis, breathing regulation and airway control (McKenna, 1986).

*Breastfeeding.* All young babies in the evolutionary context have invariably been breastfed, which can be considered a highly dense developmental experience involving motor, sensory, integrative, and social stimuli and interactions. As we have described, the breastfeeding experience also seems to revolve around the infant’s central challenge—to ensure sufficient airflow and escape suffocation. After all, while at the breast, respiration through the mouth is impossible and the nostrils are the only source of oxygen. How tightly nursing at the breast relates to airflow challenges can be seen from the evolutionary “design” of the suckling’s nose: the nares are pointed upward to yield a “button nose” that allows for unobstructed breathing at the lactating breast.

The link between breastfeeding and airway management challenges was explored more than 50 years ago by the British pediatrician Gunther (1961). Her observations showed that breastfeeding is commonly associated with episodes of airway obstruction in the infant and that these experiences reliably stimulate protective escape maneuvers on the part of the baby to find relief from smothering. These liberating maneuvers—which include side-to-side head waving, head withdrawal, and arm jabbing—may prime the infant for similar challenges encountered in other situations, including prone sleeping. Such transfer of a learned response appears plausible from experiments with nasal air-jet stimulation during sleep in which young breastfed infants arouse more readily from active sleep than formula-fed infants (Horne et al., 2004). Breastfeeding could thus be viewed as an anti-asphyxia training of sorts—and a “guided” one, too.

Along this line of reasoning it may be hypothesized that breastfed infants, being better prepared to cope with respiratory compromise, should have a lower risk of dying from SIDS in the prone position than non-breastfed infants—after all, the prone position may require superior skills of airway protection relative to the supine position. A preliminary calculation from data from the CESDI and SWISS studies (400 SIDS cases and 1385 controls) could lend support to this hypothesis; the odds ratio of being found prone in the last (respectively, reference) sleep was 2.7 [95% CI: 1.31–5.56] amongst current breastfeeders; for non-breastfeeders it was 9.97 [95% CI: 7.06–14.09] —and thus three to four times higher. Alternatively, this could be a reflection of the fact that fewer breastfed infants are put down in the prone position in the first place—for example, when bed-sharing.

*Infant carrying and extensive parent*–*baby contact.* Babies in the evolutionary context have been moved and carried by their caregivers—on their hips, in their arms, in slings, or in devices manufactured from natural materials. It therefore comes as no surprise that being carried appears as a biological adaptation in humans (Lozoff & Brittenham, 1979). This experience of constantly balancing gravity and dealing with changing environmental and social exposures presumably represents an exquisitely stimulating environment for the muscular development of the neck, legs, and trunk but also for perceptional tasks and social learning. The same holds true for other species-typical care practices such as frequently picking infants up and providing them with physical proximity, social interaction, and regulatory resonance. As a side note (and speculation uncorroborated by research as of yet), the experience of being carried may also include challenges and thus “learning opportunities” for airway control, similar to what has been described for breastfeeding: for a baby, being transported snug to the body may indeed be a constant exercise in airway control.

*Bed-sharing.* Sleep constitutes a human baby’s predominant daily behavior. In the evolutionary context, human infants have slept in close proximity to their mothers, starting from the first day of life—after all, babies placed alone for sleep would have been more liable to be abducted by predators, to be bitten or stung by reptiles and insects, or would have suffered compromised thermoregulation in most climate zones. Indeed, for the human infant, the solitary sleep arrangement represents a historically and biologically novel environment (Gettler & McKenna, 2011).

The possible maturational impact of routine and early onset bed-sharing and its protective ramifications to SIDS have been described in detail (Gettler & McKenna, 2011). When placed in proximity in the sleep laboratory, sleep stages not only tend to synchronize between the mother and her infant, but also, on the part of the baby, sleep architecture changes—breastfed, bed-sharing infants spend more time in active sleep stages than babies sleeping in their own crib during their first months of life (Gettler & McKenna, 2011; Mao et al., 2004; Mosko et al., 1996, 1997). Concomitant with the higher proportion of active sleep—marked by higher muscle tone and more frequent arousals—motor activity during sleep is higher in the bed-sharing setting than during solitary sleep. Also, during dyadic sleep, babies not only have more wake periods and more frequent feeding sessions but also experience more passive repositioning and checking for safety (Baddock et al., 2006; Ball, 2009). At the same time, bed-sharing represents a rich interactional matrix for developmental stimulation. It is therefore conceivable that bed-sharing infants, within this matrix, may more easily acquire protective regulatory competences. This may explain why routinely bed-sharing infants—those who have “practiced” co-regulatory sleep from early on—do not seem to be at an increased risk of SIDS in the bed-sharing situation when compared with infants exposed to bed-sharing without prior experience (Klonoff-Cohen & Edelstein, 1995a; Vennemann et al., 2012). Here, again, the timely acquisition of competences in airflow maintenance and airway protection may be included, even though their exact nature and mechanism are yet to be elucidated (for a complex hypothesis, see McKenna et al., 2016).

As the above discussion of the evolutionary care environment shows, the original care of the human infant, the most immature of all primates at birth, is closely interlinked with physiological regulation (McKenna, 2004). As a matter of fact, all the described components of the “evolutionary care package” represent interactional, contingent, multisensory opportunities for the rapid experience-based learning of regulatory behaviors and can therefore be regarded as built-in stimuli for maturational advancement. Growing up within the evolved frame of proximal care may indeed be viewed as an appropriately scheduled training experience to the infant pertaining to musculoskeletal, sensory, neurological, and autonomous-regulatory development. From a developmental perspective, this maturational experience could indeed be framed as “physiologic acceleration” resulting in appropriately developed protective behaviors.

## Back to Sleep: and What About the Sleeping Position?

The choice of a safe and physiologically appropriate sleeping position for the human infant should also be part of the evolutionary care package. Like the other components of this adaptive system, this “choice” should increase the infants’ fitness—in other words, support their survival.

Here, the prone position stands out as a veritable paradox: the most risk-laden infant sleep position seems to come with tangible advantages for both the infant and their caregivers. Human infants placed prone spend more time in deep sleep (Hashimoto et al., 1983; Kahn et al., 1993; Sahni et al., 2002), sleep longer, wake up less often (Amemiya et al., 1991; Brackbill et al., 1973b; Hunt et al., 1997; Kahn et al., 1993; Skadberg & Markestad, 1997), and cry less (Brackbill et al., 1973a; Keitel et al., 1960). They are reported to have lower rates of motor activity during sleep (Brackbill et al., 1973a; Hashimoto et al., 1983), to have lower energy requirements (Ammari et al., 2009; Masterson et al., 1987), and to be less reactive to noise (Franco et al., 1998) as well as to other environmental challenges such as tilt maneuvers (Galland et al., 1998) or air-jet stimulation (Horne et al., 2001). In early observations of neonates in the hospital setting where infants were alternately placed prone and supine for sleep without interventions by staff, sleep duration was 35% shorter and total crying time was 5 times longer during supine sleep relative to prone sleep (Brackbill et al., 1973a). To this day, the prone position is exploited by neonatologists to treat the most fretful of their patients—neonates withdrawing from prenatal exposure to narcotics (Maichuk et al., 1999). This “sleep promoting effect” of the prone position (as the late sleep researcher André Kahn put it; Kahn et al., 1993) may explain why healthy infants sleeping alone prefer to sleep prone rather than supine once they are able to roll over and choose their sleep position by themselves (Togari et al., 2000). And it may explain why some parents still place their infants in the non-recommended prone position—often even in spite of being professionally counseled against this practice (Brenner, 1998; Colson et al., 2017; Moon & Omron, 2002; Ottolini et al., 1999). The practical advantages of the prone position may also explain why boys—who cry more (van der Wal et al., 1998), are more easily arousable during sleep (Richardson et al., 2010), and in general pose higher demands on care than girls (Kraemer, 2000)—are being positioned prone nearly twice as often than girls (Jonge et al., 1994; Ottolini et al., 1999; Oyen et al., 1997).

Also, the prone position seems to come with positive effects on infant development. Both observational and experimental studies indicate that infants who are routinely being placed prone for sleep during the early postnatal period have more advanced gross motor development at least temporarily as compared with supine-sleeping infants (Davis et al., 1998; Dewey et al., 1998; Holt, 1960; Jantz et al., 1997; Majnemer & Barr, 2005; Mitchell et al., 1999b; Paluszynska et al., 2004; Priyadarshi et al., 2022; Task Force on Infant Positioning and SIDS, 1996; Togari et al., 2000; Vaivre-Douret et al., 2005), making some pediatricians wonder if the tests for developmental assessment validated in the pre-Back-to-Sleep era can still be used now (Schindler & Hausman, 2001).

The positional paradox also extends to the side sleep position. Being placed in this position (which carries the risk of falling prone) is reported to be associated with a higher risk of SIDS (Baddock et al., 2004, 2006; Li et al., 2003; Scragg & Mitchell, 1998). At the same time, infant sleep research describes the side position as a normal sleep situation for babies sleeping in proximity to their breastfeeding mothers—considered the species-typical arrangement in humans by evolutionary biologists as well as anthropologists (Gettler & McKenna, 2011; McKenna & Gettler, 2016; Trevathan, 1996). As these observations show, the mother frequently repositions her baby after nursing and even while she is asleep, the preferred placement for the infant being on the side and supine—in other words, in positions that facilitate easy access to the breast (McKenna et al., 1994, 1997). Indeed, breastfed bed-sharing infants apparently spend more than half of their sleep time in the side sleeping position. The lesser risk of the side position in the bed-sharing situation could be due to the fact that breastfed infants are typically held in the so-called *cuddle curl* by their mothers, which may prevent the infant from falling prone (Ball, 2006).

Not surprisingly, there is intense debate about which sleeping position may be normative for the young *Homo sapiens* (Bergman, 2015; Brackbill et al., 1973a). From an evolutionary angle, this question may not be as easy to answer for the human infant as for other primates (the latter all sleep non-supine at their mother’s bodies in infancy). For one, there is a dearth of descriptive data on infant sleep in the prehistoric past. After all, *Homo sapiens* follows very different culturally constructed norms, which in part reflect assumptions about the “right” mother–infant proximity, for instance (Ball, 2006; Jenni & O’Connor, 2005; Rudzik et al., 2021). Profound changes in sleeping routines for infants have been described with the transition from the hunter-gatherer lifestyle (“back and hip cultures”) to the agrarian subsistence pattern (“crib and cradle cultures”) (Jenni & O’Connor, 2005; Whiting et al., 2013), and these changes imply not only the sleeping position (supine vs. side vs. prone) but, very importantly, also the physical and social context in which infant sleep happens. In the hunter-gatherer context, sleeping in close proximity to a moving, sitting, or sleeping caregiver can be considered a normal experience for the human infant. This may also have implied sleeping prone—inclined prone or upright prone—on the mother’s resting or moving body, starting right from birth—in other words, in a co-regulatory context of proximal awareness, similar to what is being observed in other primates. Being placed prone alone on a solid surface for sleep, however, may not have been a normal sleep experience in an environment marked by predation. There are indeed some experimental hints that the physiologic effects of the prone position also depend on co-regulatory input, as described above.

At the same time, other sleeping positions may also have been expectable for the hunter-gatherer infant, such as sleeping on the side or on the back while sharing the same sleep surface with the mother during sleep. As observational data show, side or supine sleep seem indeed to predominate in this context (McKenna et al., 1994, 1997). Therefore, from evolutionary considerations, neither the prone position per se nor the supine position per se can be considered the “norm” for the human infant—all sleeping positions may have been typical in certain (social) contexts and thus come with qualifiers.

Yet, the evolutionary tenet remains valid for all positions: whatever the sleeping position, it should be safe—in other words, not go against the survival interest of the child. So, what may have made the evolved practice of prone sleeping “safe”? Clearly, we are on speculative grounds here since this has not yet been explored to our knowledge. One suggestion may be that early prone sleeping in a co-regulatory context—in other words, on a living body—may at the same time provide the infant with input needed for safe learning. After all, the neonate enters this experience with its innate reflective repertoire, which has been shown to be more effective in the first few weeks than a few months later. Also, he or she receives co-stimulatory and co-regulatory input, possibly similar to what has been observed during dyadic sleep in general where the mother regularly checks on the baby and intuitively tends to its signals by touching and repositioning the baby (see above).

While sleeping on a body there may even be co-regulation on a deeper physiologic regulatory level. The cuddling infant utilizes the physiologic temperature regulation system provided by the mother, drawing exactly the amount of heat from the mother’s skin that he or she needs to maintain a constant surface temperature (Ludington-Hoe, 2015). There are indications that infants also utilize their mothers’ breath signals for their own breathing regulation. This has been experimentally shown in premature infants who have been provided with a “breathing teddy bear” in their bassinets that “breathes” at a set rate reflecting the infant’s own respiration rate during quiet sleep. The babies who slept with the breathing bear had more regular, calmer sleep than children who had only a non-breathing teddy bear at their side (Ingersoll & Thoman, 1994). These experiments also showed that the babies sought contact with the breathing bear on their own during sleep—something they did not do with the non-breathing bear. The researchers therefore also assume that the rhythmic stimulation supports the children’s brain maturation (Provasi et al., 2021; Thoman et al., 1991).

So, plausibly, prone sleeping on a living surface may come with maturational support that may also help the infant rapidly “learn” how to get along with the challenges of this sleeping position (provided that the “living surface” may be able to provide intuitive support—in other words, not be incapacitated by drugs or alcohol and be intuitively primed to start with—similar to the prerequisites of a safe bed-sharing environment). However, this hypothesis is thus far not supported by evidence and therefore not suited to change any recommendations for infant care, which continue to identify the supine sleeping position as the safest option.

## The Biocultural Perspective

The role of the “evolutionary care package” as appropriate developmental stimulation may become even clearer if we relate the evolved developmental trajectory to the current infant care situation—an endeavor frequently undertaken to identify possible biocultural mismatch constellations, which may overextend or undermine the child’s adaptive (evolutionary) repertoire. Mismatch constellations can theoretically arise from environmental challenges unexpected by evolution (an example would be intensive and prolonged pacifier use, which may have lasting untoward consequences on the development of orofacial structures) or from developmental challenges unexpected by evolution (here, an example would be an infant being raised on a vegan diet).

Possible mismatch situations relating to the evolutionary-developmental theory of SIDS could arise from three sources:

- The infant’s current developmental environment, which in some instances may not provide the child with enough learning opportunities to build up its protective competencies. Indeed, the modern care environment is often marked by comparatively little stimulation, little body contact, and comparatively little interaction—and sometimes even by the entire lack of some components of the species-typical care package such as breastfeeding or being carried on the body.
- A possible mismatch situation could also arise from environmental challenges not “anticipated” by evolution: for instance, an infant having to deal with environmental tobacco smoke or sleeping solitarily in a separate room.
- A third source of mismatch could arise from neurodevelopmental risks—conditions that interfere with the appropriate development of protective competences for biological reasons. Intrauterine exposure to nicotine or other toxic substances, for instance, could constitute such neurodevelopmental risks, and so could extreme prematurity (which is certainly not a condition evolutionarily “anticipated” since the survival of extremely premature infants is a very recent cultural advancement).

The latter example may serve as a reminder that not all mismatch situations are amenable to change—an important point to be considered when it comes to discussing the ramifications of the evolutionary-developmental theory for preventive strategies.

## Summary of the Hypothesis

**Fig. 1:**
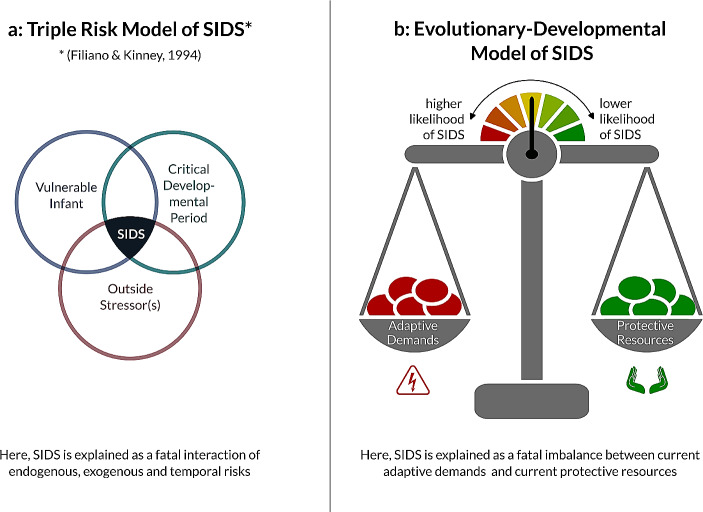
Comparing (**a**) the evolutionary-developmental model of SIDS and (**b**) the triple risk model of SIDS. While the triple risk model explains SIDS as a fatal multilevel interaction of risks, the evolutionary-developmental model views SIDS as a fatal imbalance between current adaptive demands and current protective resources. Situations with increased adaptive demands would be, for example, prone sleeping, unstable side position, heavy or loose bedding, concurrent infection, overly soft or tilted sleep surfaces, or bed-sharing under hazardous circumstances. Protective resources build up during physiologically normal infant development in a species-typical care and practice environment and reflect accumulated developmental abilities. The Venn diagram depicting the triple risk model (a) was originally published by Filiano and Kinney (1994)

To tie the pieces together into a testable hypothesis, we are claiming the following: SIDS is not sufficiently explained as a convergence of risk factors. We posit that the vulnerability to SIDS may be better explained as an imbalance between protective regulatory abilities on the part of the infant and current regulatory demands. In short, SIDS may be viewed as a developmental deficit uncovered by adverse environmental conditions (Fig. 1).

These developmental deficits may reflect biological disadvantages (such as intrauterine or postnatal growth restriction) and/or environmental disadvantages—both exposures do not allow for adequate acquisition of protective behaviors. Environmental disadvantages may arise from the mismatch between “distal” modern infant care practices and the “proximal” care exposures against which infant development has been evolutionarily calibrated.

## Testing the Hypothesis

If this evolutionary-developmental hypothesis holds true,

- The study record should show that the risk profile of SIDS can be fully explained through factors that indicate (a) increased adaptive challenges for the infant and/or (b) decreased maturational preparedness of the infant (exhibit 1).
- Studies should show attenuation of SIDS risk for lifestyle factors that are part of the “evolutionary care package” (exhibit 2).
- The SIDS rates within different populations should vary along the extent to which they practice the “evolutionary care package” (exhibit 3).
- The epidemiological trends in SIDS over the past few decades should be explainable within the evolutionary-developmental theory (exhibit 4).
- The evolutionary-developmental theory proposed should help explain the risk associations of SIDS that have so far remained unexplained (exhibit 5).

### Exhibit 1: The Study Record Should Show that the Risk Profile of SIDS is Fully Explained Through Factors that Indicate (A) Increased Adaptive Challenges for the Infant and/or (B) Decreased Maturational Preparedness of the Infant

If SIDS is indeed related to the absence of protective developmental capacities, we would expect SIDS *to happen in circumstances associated with a greater need for self-protection.* This is clearly borne out by the epidemiological record. SIDS risk increases with bed-sharing under hazardous circumstances, bed-sharing without prior experience, heavy bedding, a history of infectious symptoms, inappropriate sleep surface, unphysiological ambient temperature, use of mind-altering substances by the caregiver, current cigarette smoking by the caregiver, being placed alone in a separate room, or being placed prone without prior experience or in a hazardous environment (Lindgren et al., 1998).

At the same time, we would expect SIDS *to be associated with intrinsic or social influences that may interfere with the development of protective abilities*—in other words, with prematurity, low birth weight, intrauterine growth retardation, inadequate postnatal growth velocity, cigarette smoking during pregnancy, being raised in socially disadvantaged circumstances, or being raised by a young, single mother without support or prior experience. Again, all these influences are borne out by the epidemiological record (Oyen et al., 1997).

Interactions between the two categories support the argument: prematurity or having smoked during pregnancy, for example, have a much greater risk effect for babies who bed-share (McGarvey et al., 2006), who are being placed prone (Oyen et al., 1997), who are currently exposed to cigarette smoke (Blair et al., 1996), or who sleep in an unsafe sleep location (Blair et al., 2014).

### Exhibit 2: Studies Should Show Risk Attenuation for Lifestyle Factors that are Part of the “Evolutionary Care Package”

If protective abilities may indeed be more likely to develop through exposure to a species-typical care environment, we should see risk attenuation for SIDS with *breastfeeding, baby carrying, bed-sharing, and a generally responsive social environment.*

Unfortunately, as of yet, only data on breastfeeding and bed-sharing are available (no single study so far has correlated the practice of infant carrying—versus pram or stroller use—with the risk of SIDS).

Although breastfeeding has been clearly associated with an overall decreased risk of SIDS (Hauck et al., 2011) (see below), bed-sharing remains a contentious exposure in terms of its risk effects (Carpenter et al., 2013). This may relate to the fact that what is considered “bed-sharing” in the research record often is a far cry from bed-sharing as an infant-appropriate, species-typical developmental experience. Also, with bed-sharing, some unfavorable parental practices or attributes become more consequential than in the solitary sleep arrangement due to the closer proximity between baby and caregiver. In these unfavorable contexts, bed-sharing may indeed pose increased regulatory demands on the infant, which then may overextend the regulatory capabilities at least of the developmentally disadvantaged among them. In McGarvey and colleagues’ analysis of SIDS victims in Ireland, for instance, 87% of SIDS victims who died while bed-sharing had mothers who smoked during pregnancy (compared with only 17% of controls) (McGarvey et al., 2006). It is therefore not surprising that a protective effect of bed-sharing may so far have gone undetected. Indeed, as of yet, no analysis has been undertaken to compare truly low-risk bed-sharing (i.e., bed-sharing of infants with their breastfeeding, nonsmoking, and nondrinking mothers, in a normal bed without artificial hazards) with the solitary sleep experience.

However, a protective effect of “regular” bed-sharing on SIDS seems plausible from a vast array of observations:

- In an analysis of all studies with appropriate data, the risk of bed-sharing appears to be nonsignificant for infants of nonsmoking mothers (Fleming et al., 2015).
- The same analysis of studies with appropriate data has not found a risk association of bed-sharing and SIDS for infants over the age of 12 weeks (Fleming et al., 2015).
- For this group of older infants, bed-sharing indeed turns out to be a highly protective exposure if adjusted for only a few confounders such as cigarette smoking, alcohol use, and sleeping on a sofa (Blair et al., 2014). Indeed, in Fleming & Blair’s analysis of 179 SIDS deaths, only 1 SIDS case occurred in a nonhazardous bed-sharing environment compared with 61/714 (8.5%) of age-matched surviving control infants, yielding an OR of 0.08 [95% CI: 0.01–0.52] (Fleming et al., 2015).
- Of the two empirical studies currently available that have looked at the risk of routine bed-sharing (i.e., bed-sharing practiced as a family lifestyle) as opposed to nonroutine bed-sharing (bed-sharing during the last night only), neither showed an increased risk for SIDS for routinely bed-sharing infants (Klonoff-Cohen & Edelstein, 1995a; Vennemann et al., 2012).
- How much family lifestyles matter has been confirmed in a latent class analysis of a birth cohort of 7447 infants by Fleming and Blair in which the infant care practices of breastfeeding and bed-sharing appear to be mutually supportive and inextricably intertwined (Blair et al., 2010; Fleming et al., 2015).

Moreover, there is evidence that the experience of routine bed-sharing may attenuate both environmental and biological risk factors which are significant in the pooled statistical record:

- In all studies of SIDS, boys are at a greater risk of dying from SIDS than girls. Indeed, about two thirds of SIDS victims are male (Blair et al., 2006), a proportion seen across all age groups (Moon et al., 2007). This disparity may be explained as a reflection of an apparent higher biological risk of boys, who have been identified as the developmentally less stable and more vulnerable sex (Kraemer, 2000). In the bed-sharing population, however, boys are at a *lower* risk of SIDS: whereas the male to female SIDS ratio is 2:1 for the solitary sleepers, it is 0.8:1 for bed-sharing infants (Byard et al., 2012). Bed-sharing—which during the peak age period of SIDS is practiced more often with boys than with girls (Blair & Ball, 2004)—may provide the infant with maturational input for better development of protective competences and may thus attenuate the biological “disadvantage” of boys. (Alternatively, the bed-sharing experience may be a marker for other positive maturational influences independent of bed-sharing from which boys may benefit more than girls.) Along with the “boys as the more vulnerable sex” hypothesis, it may indeed be that boys are more dependent on the full development of protective developmental resources to buffer them against any intrinsic vulnerabilities that they may bring along.
- Prone sleeping seems to be less predictive of SIDS in routinely bed-sharing infants than in infants sleeping solitarily (Blair et al., 2014). Also, while bed-sharing infants and non-bed-sharing infants were shown to be placed prone for sleep at a similar rate, bed-sharing infants may be less likely to be found dead in the prone position (McGarvey et al., 2006).
- The evolutionary-developmental theory may also explain another, seemingly paradoxical finding. In an analysis of the SIDS cases from the New Zealand cot death study, bed-sharing was shown to be a risk factor only in the group of victims *not* found face down at the time of death—an association which persisted even after controlling for confounders, including maternal smoking (Thompson et al., 2006). The latter group (i.e., those not found face down) is marked by a much higher odds to be among those not usually placed prone for sleep, suggesting a lack of experience with sleeping under relatively more challenging conditions.

Although many of these explanations remain unproven they may indicate that exposures which are part of the “evolutionary care package” could be preventive influences against SIDS.

What about the prone position? If the assumption is correct that early-neonatal-onset, co-regulated prone sleep at the mother’s body was part of the species-typical care arrangement across the evolutionary scale, the corresponding exposure could indeed have been a protective or at least innocent exposure. However, here again we clearly need to recognize that prone sleeping in the evolutionary package was either directly monitored or came with a proximity awareness. The prone position was thus very different from today’s prone experience (for details, see “The Evolutionary Perspective,” above).

Also, uncertainties remain and need to be considered: extreme prematurity, for instance, was certainly not an issue on the evolutionary scale. Neither was—probably—exposure to cigarette smoke during pregnancy. Nor was co-regulation with a mother obtunded by medication, drugs, or alcohol. Clearly, these influences preclude any general proposition about the “safest” sleep position in today’s environments. Moreover, the assumption of co-regulatory prone infant sleep in the evolutionary context is far from being scientifically proven. Therefore, the discussion around the evolved sleeping position is bound to remain the most difficult and most contentious part of the “evolutionary care package”.

### Exhibit 3: The SIDS Rates Within Different Populations Should Vary Along the Extent to Which They Practice the “Evolutionary Care Package”

Reported SIDS rates differ widely around the globe (Davies & Gantley, 1994; Lee et al., 1989; Nelson et al., 2001; Wolf & Ikeogu, 1996). At the same time, ethnic groups around the world vary in their infant care practices, including the extent to which they adhere to the “evolutionary care package.” Unfortunately, these variations are difficult to draw conclusions from since databases on SIDS cases may not be reliable, especially in those countries with more traditional lifestyles. For example, a low incidence of SIDS has been suggested for some African countries (Keeley, 1992; Kibel et al., 2008; Wolf & Ikeogu, 1996). Yet, to our knowledge, only one prospective series exhibits case ascertainment according to international standards—from Zimbabwe, indicating a very low SIDS incidence (Wolf & Ikeogu, 1996).

The lower incidence of SIDS reported for Asian countries may be more reliable in this respect (Lee et al., 1989; Sawaguchi & Namiki, 2003). The latter association has been ascribed to a low-risk lifestyle (especially, the absence of maternal smoking or alcohol consumption). However, in Asian countries traditional infant care practices have also prevailed on a wide scale, with bed-sharing being considered normal, for instance.

Another approach may be to look at the variances in SIDS rates *in different ethnic groups within given countries.* Here again the interpretation needs to be cautious since many ethnic populations adhering to more traditional infant care practices are socially disadvantaged, so the outcomes may be influenced by the presence of specific risk factors such as premature births, cigarette smoking, or alcohol and substance abuse. A case in point may be the Maori population in New Zealand or the Aboriginal population in Australia, which still practice at least parts of the “evolutionary care package” yet have reported problems with alcohol and/or drug abuse that potentially increase the risk of hazardous bed-sharing and in general represent a higher risk profile for SIDS (Mitchell & Scragg, 1994).

Striking ethnic variations have been reported from South Africa, where cot death rates varied by a factor of 14 between the different ethnic groups, with the lowest SIDS rate reported for the Black population, which widely adheres to a more traditional lifestyle with infant carrying, breastfeeding, and co-sleeping (Kibel et al., 2008). However, here again, the interpretation may be difficult due to possible differences in the risk profile and in classifying and reporting SIDS between the ethnic groups.

Fortunately, comparative data exist for ethnic groups in which the risk environment is well described and ascertainment of death causes may not be biased. For England and Wales, a nearly fivefold variation in SIDS risk was reported for the different ethnic groups, with the lowest risk for Indian, Bangladeshi, Pakistani, white non-British, and Black African babies (Balarajan et al., 1989; Kroll et al., 2018; Kyle et al., 1990). This is remarkable because these families very frequently live in deprivation areas with constrained resources. Also of note, the variations described are not explained by differences in preterm birth, baby’s sex, maternal age, or area deprivation, suggesting cultural factors as explanation (Kroll et al., 2018). While the latter may include lower lifestyle risks in the respective communities, the authors point to the fact that “protective infant-care traditions may tend to persist in ethnic groups with high proportions of mothers born outside the UK.”

This is supported by two other studies which found that Pakistani and Indian parents were more likely than white British parents to report that their babies bed-shared (Ball et al., 2012; Farooqi et al., 1991) and that Bangladeshi babies were less likely to be alone at any time and would remain with adults during daytime sleeping (Gantley et al., 1993). In general, the care environment of the Asian populations in question is described as stimulatory, with babies frequently being picked up and interacting intensively: “Throughout the day the extended family network provides an almost constant source of contact with adults and other family members” (Davies & Gantley, 1994).

This reference to an engaging traditional lifestyle may also explain an interesting association reported from the United States: the longer Asian families lived in there, the higher was the risk of SIDS for the infants in these families (Grether et al., 1990). The same team had previously described that the incidence of SIDS among infants of Chinese immigrants in California was between 5 and 38 times higher than the incidence reported for ethnic Chinese in Hong Kong (Grether & Schulman, 1989).

Also, there could be merit in comparing parenting subcultures. In many Western societies, distinct parenting subcultures have formed around the idea of raising infants more “naturally” (i.e., within the framework of the “evolutionary care package”). Unfortunately, to date, no data exist from which to derive an estimate of the SIDS incidence specific to this select population. Inferring from the risk reduction adherent to breastfeeding (and possibly to bed-sharing under nonhazardous circumstances), the risk should be very low. Yet, with respect to the mostly low-risk lifestyle practiced in these subcultures and possibly a lower rate of concomitant gestational risks, it would be hard to tell if the low incidence was due to higher developmental resources of the infants in this group or to lower environmental risk factors for SIDS. Future research may shed light on this question, especially if all markers of a more proximal parenting style are considered, including baby carrying.

### Exhibit 4: The Epidemiological Trends in SIDS Over the Past Few Decades Should be Explainable Within the Evolutionary-Developmental Theory

During the 1990s, SIDS incidence decreased on a large scale, followed by smaller decreases thereafter in most countries. This may be best explained by the surprisingly fast transition to supine sleeping in the population during these years, even if this may not be the only factor (Einspieler et al., 1997; Wennergren et al., 1997). The strong preventive effect of the Back-to-Sleep advice offered, among others, by the National Institutes of Health (US), is easily understood from a developmental perspective: the supine sleeping position seems to pose less regulatory demands on the human infant and thus may confer survival benefits to the subset of infants with developmental challenges (be they biological or environmental). Practically speaking, beginning with the launch of the Back-to-Sleep campaign in the early 1990s, more and more of the infants at increased risk of SIDS due to developmental vulnerability now survived as they were placed in the physiologically less challenging (supine) sleep position.

The still visible, yet smaller decrease in SIDS incidence since the start of the millennium may be explained by the fact that several environmental risk factors for SIDS have continued to decrease since then, notably smoking around babies, overheating, and avoiding head covering, soft mattresses, and heavy bedding.

With the major environmental risk factor, prone sleeping, partially eliminated during the 1990s, the relative contribution of the developmental determinants of SIDS may have increased in importance since then (Pollack & Frohna, 2001; Trachtenberg et al., 2012). In a comparison between SIDS cohorts from 1983 to 2003, the proportion of preterm infants has increased from 12 to 34%, exposure to intrauterine nicotine has increased from 57 to 86%, while 50% fewer SIDS victims were breastfed in 2003 than in 1983 (Blair et al., 2006). All three factors may be seen as indicators of fewer developmental resources in the 2003 SIDS cohort.

### Exhibit 5: The Proposed Evolutionary-Developmental Theory Should Help Explain the Risk Associations of SIDS That Have so Far Remained Unexplained

There are still quite a few white spots in SIDS research—associations picked up in case-control studies that are not well explained by the current understanding of SIDS. In the following paragraphs we show how the developmental perspective may help explain some of these gaps.

## Outlook: Could the Evolutionary-Developmental Theory Resolve Other Open Questions in SIDS Research?

### The Breastfeeding Riddle

The protective effect of breastfeeding in relation to SIDS remains unexplained. Could this effect be mediated by an immunological advantage? Maybe, but so far SIDS has not been understood as an immunological disorder. Could breastfeeding be just a marker for an intrinsically lower risk environment? The protective influence of breastfeeding persists when adjusted for known risk factors, but of course as yet unmeasured environmental factors could be related to breastfeeding, to SIDS, or to both (Hauck et al., 2011; Vennemann et al., 2009).

The protective effect of breastfeeding may obtain clarification from a developmental perspective: breastfeeding may either be a marker for a developmentally appropriate care environment or an experience that in and of itself promotes protective developmental capacities. Both explanations seem compatible with the study record. Breastfeeding seems to be a reasonable predictor for routine bed-sharing and vice versa (Blair et al., 2010), so breastfeeding may in part be an indicator of an otherwise “developmentally enriching” lifestyle. Indeed, breastfeeding seems to be doubly linked to the practice of bed-sharing. For one, bed-sharing is associated with a higher likelihood of an infant to be breastfed; also, breastfed infants are nursed more frequently in the bed-sharing setting than when they sleep alone (McKenna et al., 1997). Also, even if they are not bed-sharing, breastfed babies may be more likely to sleep in the parents’ room instead of being moved to a separate room. Likewise, breastfed babies, by virtue of their typically more closely spaced feeding intervals, may have more maternal “supervision” and thus checking for safety. This may fit well with the observation in one study that breastfeeding seems to confer protection only for the solitary sleepers (who, by virtue of the more frequent “visits” by their mothers, now may attain some degree of co-regulation) (Blair et al., 2014). Supportive of this explanation could be the observation that the protective effect of breastfeeding may be greater with longer duration of breastfeeding (Thompson et al., 2017). However, other studies have not seen this effect and report protective effects for breastfeeding of any duration (Hauck et al., 2011).

An intriguing developmental explanation already mentioned above pertains to the fact that infants seem to practice protective behavior pertaining to airway and breathing control while at the breast as newborns (Gunther, 1961). According to these observations, the breastfeeding experience may represent an exquisite learning environment for the expansion and augmentation of inborn reflexes involved in breathing and airway control, which in turn may enhance autonomic control and arousal management in general. The apparently very effective learning at the breast may explain why any breastfeeding (i.e., without duration specified) was associated with roughly the same protection as breastfeeding beyond 2 months of life (Hauck et al., 2011) and why some epidemiological studies do not see a difference in SIDS risk reduction between fully and partially breastfed infants (Thompson et al., 2017; Vennemann et al., 2009).

Also, the finding that breastfeeding may only confer a risk reduction for those infants who sleep alone (Blair et al., 2014) may be explained by the developmental assumption. Perhaps breastfeeding in the routinely bed-sharing population loses its significance as a developmental marker or developmental “booster” because routinely bed-sharing infants, in their majority, are exposed to several components of the evolutionary care package. Or maybe infants sleeping alone may be even more dependent on some maturational support to meet the challenges of sleeping alone (i.e., without co-regulatory support).

### The Bed-Sharing Riddle

No single analysis of the risk of SIDS in infants bed-sharing under truly “infant appropriate” conditions has so far been done—in other words, in infants who share a firm, straight sleep surface with their breastfeeding, non-obtunded mother from the start of their lives. Extrapolating from the studies on routine bed-sharing (no increased risk for the infant) (Klonoff-Cohen & Edelstein, 1995a; Vennemann et al., 2012), bed-sharing in the absence of smoking (no significantly increased risk for the infant) (Blair et al., 1999; Fleming et al., 2015), as well as bed-sharing with some hazards, such as sofa-sharing, smoking, and alcohol use, removed (no increased risk for infants under 3 months of age, significant protective effect for infants over 3 months of age) (Blair et al., 2014), it appears at least plausible that bed-sharing under truly “infant appropriate” conditions may confer a *protective* effect against SIDS.

But through which mechanisms should normal, infant-appropriate bed-sharing confer protection? Several hypotheses appear convincing. For one, the effects of bed-sharing and breastfeeding are difficult to disentangle since bed-sharing clearly goes along with an enhanced breastfeeding experience in frequency, exclusivity, and duration. Bed-sharing, if one will, may help infants harvest the benefits of breastfeeding to a fuller extent. Also, “normal” bed-sharing may be a marker for a developmentally appropriate care environment in a broader sense, with other developmental stimuli included. This may explain the findings of lower SIDS risks in routine versus ad hoc bed-sharing and is also supported by the lower SIDS risks in certain countries and ethnic groups where bed-sharing is common and SIDS rates low (see “Exhibit 3” above). In addition, or alternatively, the lighter sleep architecture, the more frequent arousals and greater co-stimulatory support during bed-sharing may confer protection (Mosko et al., 1997). The latter may especially benefit infants with preexisting deficiencies in arousal or autonomic control in general (McKenna, 2004). Furthermore, the intensive social interaction which bed-sharing brings along may be considered an extended opportunity for practicing regulatory behaviors—and here again competences in airflow maintenance and airway protection may be included.

### The Riddle Around Maternal Smoking

Another major environmental risk factor for SIDS, maternal smoking, is still far from being understood, and this is true both for postnatal smoking as well as for smoking during pregnancy, whereby in its risk effect the latter by far exceeds the risk of the former (Mitchell & Milerad, 2006).

The risk effect of smoking has been related to possible apneic events or diminished arousal in infants of smoking mothers. However, at least when it comes to explaining the increased SIDS risk of infants in the prone position, this interpretation seems to be at odds with the experimental record: observed apneic events seem to be associated with an increased risk of SIDS in supine sleeping infants only (Mitchell & Thompson, 2001). Also, while infants of smoking mothers were indeed shown to have a decreased arousal response relative to the infants of non-smokers, this is only true for supine sleep and not for prone sleep (Horne et al., 2002).

So here again, the evolutionary-developmental theory may provide additional insight: infants of smoking mothers may be at a biological disadvantage when it comes to developing appropriate protective regulatory and motor abilities. A plausible explanation may start from the influence of nicotine in terms of immediate effects on neuromuscular control and/or long-term effects on infant development: as a known muscle relaxant and central regulatory depressant, nicotine may temper the current protective abilities of the infant, including its response to stress (McCormick et al., 1991; Page & Valentino, 1994)—an infant under the influence of nicotine may just be less able to mount protective countermeasures if need be. This may explain why cigarette smoking was shown to be more hazardous for bed-sharing than for solitary sleeping infants (Carpenter et al., 2004).

A developmental explanation may also be valid for smoking during pregnancy. Chronic postnatal and especially prenatal nicotine exposure was shown to go along with stunted biological maturation, including abnormal general movements at two and four months of corrected age (Bouwstra et al., 2009; Kelmanson, 1996b; Mitchell & Milerad, 2006; Schellscheidt et al., 1998). This may set the exposed infant at a developmental disadvantage, possibly extending to the acquisition of protective regulatory competences. This is ever more plausible as neuropathology studies show that intrauterine nicotine exposure also affects autonomic cardiorespiratory control (including defects in the gasping mechanism that initiates auto-resuscitation) (Dergacheva et al., 2010). This again would explain why prenatal exposure to nicotine has a much greater risk effect for babies in situations of higher regulatory demand, such as bed-sharing (McGarvey et al., 2006) or prone sleeping (Oyen et al., 1997).

### The Riddle Around Sleeping in a Separate Room

Why should babies sleeping in their own room be at an increased risk of SIDS, as the epidemiological record shows? If the risk argument is being followed, a separate room should be an exquisitely low-risk space for SIDS to happen. After all, most of the acknowledged SIDS risks emanate from untoward parental behaviors, most of which clearly exert their effects through physical proximity—such as smoking, consumption of alcohol or drugs, or overlaying. Indeed, from a pure risk perspective, the safest place for an infant should be as far away from the parents as possible. Yet, sleeping in a separate room turns out to be associated with a substantial SIDS and also suffocation hazard (Parks et al., 2022; Tappin et al., 2005).

Here again, the explanation may be found on a different plane. For one, sleep in social isolation may represent an increased physiologic challenge. From an evolutionary standpoint this is plausible. After all, in this sleep arrangement the infant’s regulatory mechanisms pertaining to breathing, thermoregulation, and arousal must function in an environment “for which they were not designed by evolution” (McKenna,^2004^). Indeed, sleeping devoid of co-regulatory support may represent a biocultural mismatch situation.

Of course, distal sleep location could also be a marker for distal infant care practices—in other words, for care practices containing little developmental stimulation and learning opportunities for the infant. Indeed, infants who have died of SIDS while sleeping in a separate room are more likely to have rolled into the prone position if they had been placed on their sides for sleep, possibly indicating lesser protective abilities (Blair et al., 2006a, b).

## Discussion

Why do some babies suddenly and unexpectedly die in their sleep without a tangible cause? Traditionally, the search for an answer has been from a risk perspective, based on descriptive analyses of the circumstances in which these tragic events happen. However, the explanatory power of the risks thus identified—such as prone sleeping, overheating, or sleeping on an unsafe surface—is limited: the vast majority of infants with an identical set of risk factors do not die of SIDS.

This hiatus and the pursuit for potential causal mechanisms has inspired researchers to examine the role of potentially protective factors. Here, the factor most robustly associated with a reduced risk of SIDS has been the experience of being breastfed (Hauck et al., 2011). In recent publications, bed-sharing under nonhazardous circumstances emerged as another potentially protective factor for infants 3 months or older (Blair et al., 2014). Also, there is some evidence of a negative risk association for the use of pacifiers and for prenatal care, but here questions remain around the causality of these influences. Regardless of these and other open questions, none of the potentially protective influences has so far been conclusively explained as to *how* they should provide protection.

The same lack of a conclusive and accepted explanation is true for the most salient feature of SIDS—its relative sparing of the neonatal period, although hypotheses have been formulated. Indeed, in a fundamental contrast to almost all other causes of infant mortality, SIDS is noteworthy for its much lower prevalence in the most immature of infants, the neonates.

In this article, we seek clarification by exploring SIDS from an extended developmental and evolutionary perspective which we call *evolutionary-developmental theory of SIDS.* Our review starts from the basic tenet of developmental biology that the developmental trajectory is a balancing act between self-expansion and self-protection: at any time, the developmental process should provide the human infant with sufficient protective resources to meet the typical challenges of the current developmental period. Based on this assumption and in reference to earlier work by Myrtle McGraw, Barbara Burns, and Lewis Lipsitt (Burns & Lipsitt, 1991; Lipsitt, 2003), we suggest that infants may be rendered vulnerable to SIDS if they cannot build up enough protective resources in the course of their development—either because they are subject to biological constraints or because they grow up in a care environment not conducive to the learning of protective competences. According to this concept, the pathogenetic matrix of SIDS may be viewed as a mismatch between the protective developmental resources on the part of the infant and the current physiological challenges he or she is faced with. In a radical take of this concept, SIDS may be described as a consequence of developmental deprivation uncovered by unusual environmental challenges. (See the Electronic Supplementary Material for a graphical summary.)

### Understanding Vulnerability

For a better understanding of infant vulnerability, we have reviewed the early experimental record on infant development which describes why and how human infants need to actively “learn” protective skills as they mature (Burns & Lipsitt, 1991; Lipsitt, 2003; McGraw, 1963). In the first few weeks of life, protection of vital physiological functions is provided by an inborn pattern of very effective reflexes which, for instance, secure airway control and autonomous regulation. These reflexes can be seen as a preinstalled shield under which human infants are safe against the typical challenges of the first leg of their precocious existence. However, as these reflexes gradually subside, the infants need to actively build up secondary, volitional abilities with which to augment, expand, and finally replace their “preinstalled” protective system. If this transition is not running smoothly, the infant is left vulnerable to external threats.

For an adequate transition from the neonatal “reflective” phase to a fully functioning volitional protective repertoire, two prerequisites must be met: first, the infant needs to be equipped with adequate developmental readiness—in other words, must be able to rely on a normal biology—an influence that may, for instance, not be a given in very premature infants, in cases of intrauterine growth restriction, and/or in infants whose mothers have smoked during pregnancy. Second, the infant needs to grow up in an environment sufficiently conducive to active, experience-based “learning” (Lipsitt, 2003).

### Understanding Protection

Which environment is needed for this learning to be successful? Here we make recourse to evolutionary theory, which states that adequate learning should happen if infants are subject to the species-typical experiences. After all, their developmental trajectory is calibrated against the experiences that human infants have made along the evolutionary time scale. Within this evolved social and physical frame, the infant should acquire enough protective skills to cope with the expectable environmental risks. This evolutionary care package includes being breastfed, sleeping in a social regulatory context, being carried, and generally growing up in a context of physical proximity, social interaction, and regulatory resonance. Any gross deviation from this experiential framework (which we call the “evolutionary care package”) may set the infant up for a possible lag in the acquisition of secondary protective skills. As we lay out in this review, these “evolutionarily expectable” exposures may indeed be the pieces of protection relevant to SIDS.

Lack of breastfeeding, for instance, was consistently shown to make infants more vulnerable to SIDS. As early observations show, breastfeeding may indeed be an opportunity for learning of effective respiratory defense responses. For example, brief events of airway obstruction while sucking at the breast can trigger rapid associative learning of more complex airway control strategies which may be utilized in other situations of airway compromise, such as while sleeping in a hazardous environment (Gunther, 1961; Lipsitt, 2003).

We have similarly investigated the bed-sharing situation and contend that this may also be a highly interactional experience for the infant supportive of the acquisition of secondary regulatory and protective skills—at least if bed-sharing happens under species-typical, nonhazardous circumstances. The epidemiological record indeed indicates that bed-sharing may be protective of SIDS for infants of 3 months or older, if only a few exposures—maternal drinking, smoking, and sleeping on a sofa—are adjusted for (Blair et al., 2014).

### Prone Sleeping: A Contentious Exposure, but Possibly Fertile for Hypothesis Generation

A large portion of this work covers the prone sleeping position so central and important to SIDS research and prevention. While it is evident that avoidance of the prone position is one of the most effective preventive measures against SIDS, it remains unclear how this effect may be explained.

Here, several observations and theoretical facets may need to be reconciled. There is evidence that the prone sleeping position may pose greater physiologic and regulatory challenges to the infant than the supine position, which may explain the higher risk of SIDS in this position (Chiodini & Thach, 1993; Galland et al., 2002; Horne, 2018; Yiallourou et al., 2008). The higher regulatory demand in the prone position may explain why infants with developmental impairments (such as intrauterine growth restriction, prematurity, or intrauterine exposure to toxic substances) seem to be at a much higher SIDS risk from sleeping prone than infants without such risks (Oyen et al., 1997). There is also evidence that neonates and younger infants in general, while still at risk, may be more resilient to the prone position than infants in the 10- to 24-week age bracket, as indicated by the clearly age-dependent SIDS risk associated with being placed in this position (Oyen et al., 1997). We refer to an extensive body of experimental work which may explain this observation.

Also, experience with prone sleeping seems to modify the risk of prone sleeping: experienced prone sleepers were shown to have a much lower SIDS risk than inexperienced ones (L’Hoir et al., 1998; Mitchell et al., 1999b; Oyen et al., 1997), with two studies reporting a nonsignificant risk for infants routinely placed prone for sleep (Klonoff-Cohen & Edelstein, 1995b; Li et al., 2003). We also present novel preliminary findings according to which breastfed infants may be at a lower risk of dying from SIDS in the prone position than bottle-fed babies (see “The Evolutionary Perspective,” above).

While the advice for general supine placement of infants clearly needs to remain the backbone of SIDS prevention, there is still a need from a public health standpoint to identify factors which may provide infants with resilience against the higher physiological challenges of the prone position. After all, the protection provided by sleeping supine inevitably ends once the infants are able to roll over. Now, they need to rely on the absence of further environmental risks (such as soft mattresses, pillows, heavy bedding, or an obtunded adult next to them), and they also need to rely on the protective resources that they may or may not have built up during their development. Our evolutionary-developmental theory provides hypotheses as to the possibly supportive and hindering factors that may be relevant for this process, such as intrauterine exposure to tobacco smoke, but also everyday experiences such as being breastfed, being carried, and being handled in close, responsive proximity. It would be helpful to conduct further analyses on existing data to verify these suggestions and identify protective influences that may make inadvertent prone sleeping—inevitable to happen at some point in an infant’s life—safe(r). Our preliminary analysis of the effect of breastfeeding could indicate some risk attenuation through this exposure.

A second issue starts with a paradox familiar to many pediatricians faced with parents who report that their infant “sleeps better” when placed prone (Brenner, 1998; Colson et al., 2017; Moon & Omron, 2002; Ottolini et al., 1999). There are indeed indications that the prone sleep position, despite its risks, can go along with some positive behavioral effects relevant to everyday caregiving, including shorter sleep latency, waking up less often, longer sleep duration, and less crying (Amemiya et al., 1991; Brackbill et al., 1973a, b; Hunt et al., 1997; Kahn et al., 1993; Keitel et al., 1960; Skadberg & Markestad, 1997). We have attempted to explain this paradox from our evolutionary-developmental theory, including whether the prone position may have been part of the “evolutionary care package.” As it turns out, there is no general answer to this question. In the hunter-gatherer context, the typical sleeping situation during nighttime sleep was a social one, next to a nursing mother. In this co-regulated context, infants usually sleep in a body position conducive to breastfeeding—in other words, on their sides or backs (Ball, 2006). Prone sleeping presumably happened in other care situations—for example, while being carried or while the caregiver was awake and engaged in other activities. Again, here, the prone sleeping situation was presumably not a solitary arrangement but rather happened—as in other primate species—within a close social regulatory context, with the infant sleeping at or on the body of an intuitively prepared caregiver. This “social” prone sleeping was presumably introduced right from the start of the infant’s life.

Prone sleeping as typical today (with the infant sleeping alone or devoid of close physical proximity and supervision by the breastfeeding mother) was presumably not part of infant care in the human evolutionary context. In other words, being placed prone for sleep flat on an inanimate surface without close supervision cannot be considered species-typical in the predator-ridden environments of our evolutionary past and is also not observed in other Great Ape species.

It appears compatible with the evolutionary-developmental perspective that the evolutionary-standard, co-regulative experience of “social prone sleeping” starting in a relatively protected state of fully developed neonatal reflexes may have provided the infant with learning opportunities to acquire volitional protective abilities and cope with the challenges of the prone situation in developmental stages to come. Again, while this may have been an inbuilt and safe process in the evolutionary scale, there are good reasons to assume that this may not apply to current cultural contexts and certainly not to all infants—if only for the fact that infants with profound developmental risks such as severe prematurity may not have survived the newborn period in the hunter-gatherer context. So, clearly, these theoretical considerations are by no means suited to draw practical advice from or dismiss current sleep recommendations. The only feasible and ethically responsible way for developmental support we see at this point with regard to the prone position is supervised awake “tummy time,” which has been shown to provide developmental benefits (Hewitt et al., 2020).

### How Does the Evolutionary-Developmental Theory Fit with the Epidemiological Record on SIDS?

We have tested our assumption that the risk of SIDS may be indicative of inadequate protective resources against the epidemiological record on SIDS (exhibits 1 through 5). Here, we find that the epidemiological record may indeed be explainable within the hypothesized model. From this analysis it appears plausible that lack of biological or experiential protection may be a powerful determinant and descriptor of SIDS.

### Open Questions for Future Research

From our survey of the literature, we also note that relatively simple questions which appear urgent from an evolutionary-developmental perspective remain unanswerable for lack of data. For instance, after more than 50 years of SIDS research, we still do not know if appropriately developed infants are at risk of SIDS at all. The data available only provide rough hunches. In a typical cohort of SIDS victims, hardly any infant can be found with no developmental risk factors—for instance, one who was *not* born to a mother who smoked during pregnancy, was *not* born premature or small-for-date, or was *not* deprived of the breastfeeding experience. Data focusing on this group of presumably “well-protected” infants are missing entirely. What is the SIDS risk of a healthy term infant born to a nonsmoking mother who breastfeeds at least in the neonatal period and who bed-shares with her infant from birth under nonhazardous circumstances? We do not know. And because we don’t, we tend to declare in our public health messages *all* infants at risk of SIDS—based on the fact that external risk factors relevant to SIDS in case-control studies are to be found in any family and can hardly be completely eliminated. But is this message correct? We do not know.

### How to Avoid Unintended Consequences

This lack of finer-grained data may also tie into a serious question that has already been entertained in SIDS research around the bed-sharing question (Blair et al., 2014). There is evidence that a general advice against bed-sharing may “backfire” in some cases, inadvertently resulting in infants dying of SIDS. This may, for one, happen because some mothers, in their attempt to avoid the bed-sharing situation, may seek unsafe places for breastfeeding (such as sofas or armchairs) and thus subject their infants to a high risk of suffocation or rolling in the prone position and dying of SIDS. Also, some mothers may be discouraged from breastfeeding and thus withhold a protective experience from their baby—again inadvertently increasing its risk of SIDS, and other health risks. Third, but very urgently to be discussed from an evolutionary-developmental understanding of SIDS, some families may miss out on the protective effects against SIDS that bed-sharing under safe conditions may bring along (not to speak of other developmental and psychosocial advantages that some families may derive from bed-sharing). All this may explain why it is paramount and in the vital interest of families to find as specific advice for SIDS prevention as possible.

It is our hope that the theoretical concept that we have presented may provide some foundation for this endeavor.

## Electronic Supplementary Material

Below is the link to the electronic supplementary material.

Supplementary Material 1

## Data Availability

Not applicable.

## Footnotes

1 Dr. Raring, whose own child died of SIDS, was an early advocate for SIDS research.
