# Correction: Death from Failed Protection? An Evolutionary-Developmental Theory of Sudden Infant Death Syndrome

**DOI:** 10.1007/s12110-025-09491-z

**Published:** 2025-04-21

**Authors:** Herbert Renz-Polster, Peter S. Blair, Helen L. Ball, Oskar G. Jenni, Freia De Bock

**Affiliations:** 1 Division of General Medicine, Center for Preventive Medicine and Digital Health Baden- Württemberg (CPD-BW), University Medicine Mannheim, Heidelberg University, Mannheim, Germany; 2 Department of General Pediatrics, Neonatology and Pediatric Cardiology, University Hospital Düsseldorf, Heinrich-Heine-University, Düsseldorf, Germany; 3 Centre for Academic Child Health, Population Health Sciences, University of Bristol, Bristol, UK; 4 Department of Anthropology, Durham Infancy & Sleep Centre, Durham University, Durham, UK; 5 Child Development Center at the University Children’s Hospital Zurich, University of Zurich, Zurich, Switzerland

**Correction to: Human Nature (2024) 35:153–196**

10.1007/s12110-024-09474-6

The Supplementary file does not have a source or copyright attribution; it has now been replaced with the correct file.

The original article has been corrected.

## Electronic Supplementary Material

Below is the link to the electronic supplementary material.

Supplementary Material 1

