# Supplementary figures and images for: Death from Failed Protection? An Evolutionary-Developmental Theory of Sudden Infant Death Syndrome
Source: Hum Nat. 2024 Jul 29;35(2):153–96. doi: 10.1007/s12110-024-09474-6 (PMC11317453; doi:10.1007/s12110-024-09474-6)

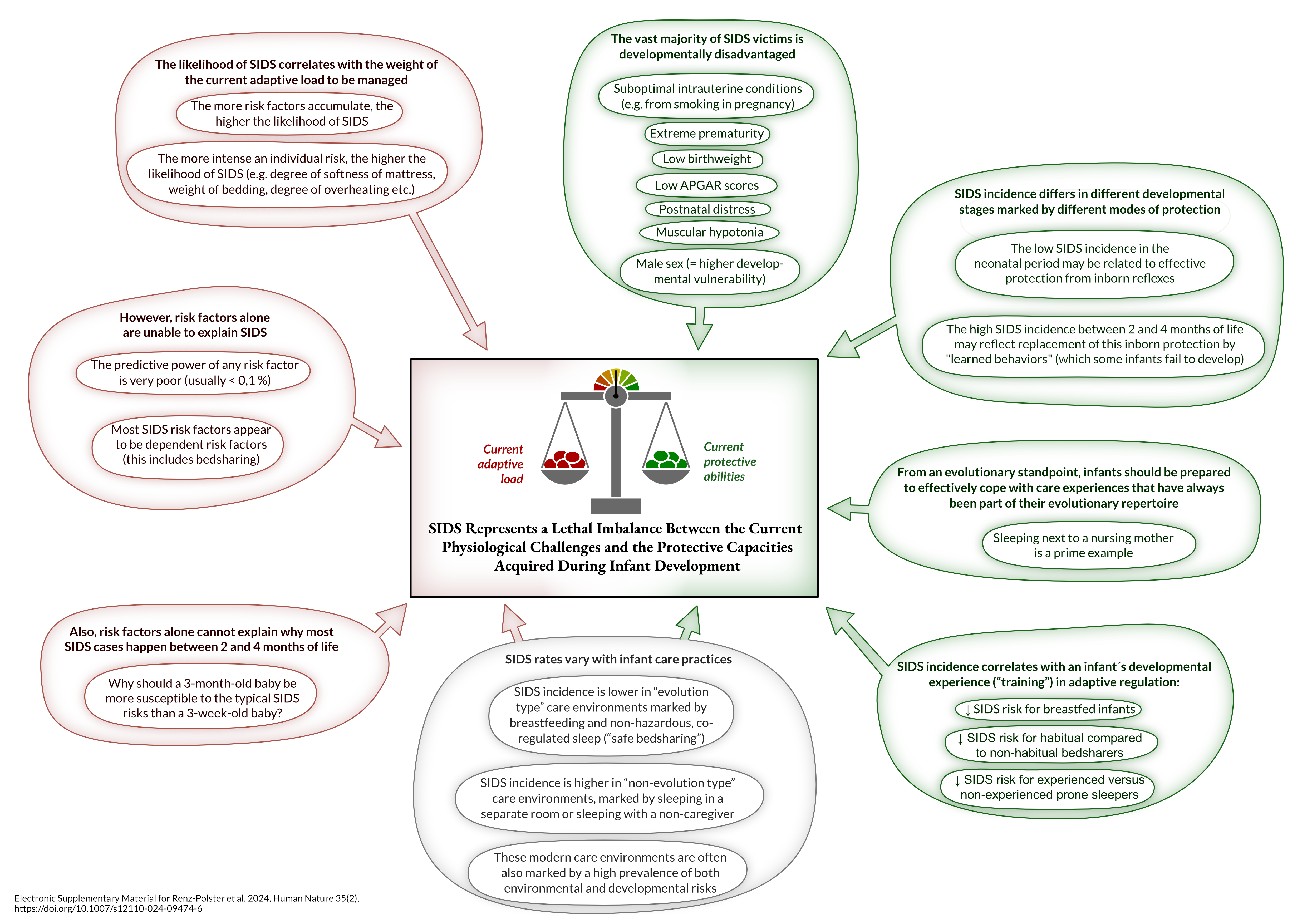

Supplement: Supplementary file 1 — Supplementary Material 1 [file 12110_2024_9474_MOESM1_ESM.png]
